# The Sense of Commitment in Individuals With Borderline Personality Traits in a Non-clinical Population

**DOI:** 10.3389/fpsyt.2018.00519

**Published:** 2018-11-06

**Authors:** Jinnie Ooi, Anna Francová, Marcell Székely, John Michael

**Affiliations:** ^1^Department of Philosophy, University of Warwick, Coventry, United Kingdom; ^2^Department of Cognitive Science, Central European University, Budapest, Hungary

**Keywords:** borderline personality traits, commitment, joint action, coordination, social expectations

## Abstract

This is the first study to test the hypothesis that individuals' sense of commitment in joint activities and relationships may be influenced by personality traits characteristic of borderline personality disorder (BPD). This study consisted of 3 online experiments implemented via Amazon Mechanical Turk. Participants were presented with videos (Experiment 1) or vignettes (Experiments 2, 3) describing situations in which everyday commitments were violated. Participants then reported their perceptions, interpretations, and affective and behavioral responses to those situations. Participants' BPD traits (BPDt) were assessed using the short form of the Five-Factor Borderline Inventory on the basis of which they were divided into two groups: High and Low BPDt. The results revealed that participants with High BPD traits were less optimistic about others acting in accordance with an implicit sense of commitment (Experiment 1), although there was no difference between groups when the commitment was explicitly stated (Experiment 3). Participants in the High BPDt group also reported heightened emotional responses (Experiments 1–3) and less adaptive behavioral responses (Experiments 1, 3) to perceived or anticipated violations of commitment. Our findings suggest that high levels of BPD traits may give rise to a difficulty in adapting one's social expectations and behavior in light of interpersonal commitments and in a manner that is calibrated to the social norms in the community. Future research should investigate to what extent a disturbed sense of commitment may contribute to the difficulties in interpersonal functioning experienced by many individuals with a clinical diagnosis of BPD.

## Introduction

In order to cultivate and maintain healthy social relationships, it is important to be proficient in prioritizing, keeping track of, and responding appropriately to our own and others' commitments. This can be especially challenging in cases in which commitments arise in the absence of any explicit agreements. Consider the following example: Roger often volunteers as an assistant at a local retirement community. One of the residents, Patricia, is celebrating her birthday today. Roger was not explicitly invited, but he knows that Patricia would be delighted if he dropped by, and that the other people involved could use his help organizing the party. He is not explicitly committed to anyone, but he may nevertheless have an implicit sense that he is committed, and this may motivate him to attend the party and to help out anyway. If so, he may also need to make various further subtler judgments as well: should he attend the party even if it would require him to decline some other important or enticing opportunity? How long should he stay at the party? Such cases are highly common, and it is important to be able to prioritize, keep track of, and respond appropriately to our own and others' commitments in cases like that of Roger.

As a starting point for investigating how people achieve this, Michael et al. (1) have recently introduced a framework based upon the notion of a sense of commitment. The sense of commitment is hypothesized to be a mechanism which identifies situations in which some other agent expects one to perform a particular action, which is a contribution to a goal of that second agent or to a shared goal, and in which that second agent is relying on one to perform that action. The sense of commitment then boosts one's motivation to perform the action that the second agent is expecting and relying on, and to resist distractions and tempting alternatives.

This framework differs in several ways from earlier proposals for conceptualizing commitment. Most importantly, earlier proposals have typically understood commitment in normative terms: as a relation among a committed agent, an agent to whom the commitment has been made, and an action which the committed agent is obligated to perform because she has given an assurance to the second agent that she will do so, and the second agent has acknowledged this under conditions of common knowledge (2–5). For example, Susie has an obligation to Jennifer to pick up the kids from school because she (Susie) has expressed her willingness to do so, and Jennifer has acknowledged this. In the canonical case, the expression is effectuated by means of the speech act of promising. The framework offered by Michael et al. (1), in contrast, is designed to illuminate the cognitive and motivational mechanisms that lead people to feel and act committed, and to expect others to feel and act committed–irrespective of whether it would in fact be justified to attribute normative obligations to anyone. This is important insofar as there are many cases (like Roger in the example sketched above) in which people feel and/or act committed, even though it is not clear whether they are under any normative obligation to do so. A second important difference is that, while earlier proposals have been tailored to cases of explicit commitment (where a promise or some other verbal assurance has been given and acknowledged), this framework is sufficiently broad to encompass cases of implicit commitment (such as the case of Roger). Making a promise is one efficient way to generate expectations and to invite others to rely on those expectations. But (as the case of Roger illustrates) expectations can also arise in the absence of any verbal assurances, as can the motivation to meet those expectations.

Working within this framework, Michael et al. (6) report evidence that a high degree of spatiotemporal coordination within joint action can give rise to a sense of commitment, leading agents to remain engaged in the joint action for a longer time and making them more likely to persist until the goal is achieved. This is because an agent's performance of her contribution within a highly coordinated joint action may provide a cue to her expectations about the other agent's upcoming actions, as well as her reliance upon those expectations. This may generate social pressure on the other agent to perform her contribution in order to avoid disappointing the other's expectation and wasting her efforts. In a similar vein, Székely and Michael (7) found evidence in support of the hypothesis that the perception of a partner's effort elicits a sense of commitment to joint action, leading to increased persistence in the face of a temptation to disengage. This is because the partner's investment of effort may provide a cue that the continuation of a joint action is likely to be valuable to her, and that she may therefore be annoyed if one disengaged.

Crucially, however, a sense of commitment that detects and responds to such cues will only be efficacious in coordinating agents' motivations and expectations about each others' actions if it is calibrated in a sufficiently uniform manner within a social group. For example, if Roger and his colleagues diverge in their sense of what constitutes a good excuse for skipping the party, or of what factors are relevant in assessing the level of commitment that is appropriate, then there is a risk that someone's expectations will be disappointed, which could threaten the harmony of their relationship. This raises the possibility that individuals whose intuitive sense of commitment is not well calibrated to their social group may find themselves frequently experiencing surprise and/or annoyance over others' failures to meet their expectations, and that their behavior may frequently be interpreted by others as evincing over- or under-commitment. In the present study, we investigated the conjecture that personality traits characteristic of borderline personality disorder (BPD) may give rise to such disturbances of the sense of commitment. This conjecture is motivated by the observation that BPD is associated with difficulties in issues related to commitment—i.e., conflicted relationships, difficulty trusting others, fear of abandonment, and patterns of overinvolvement/withdrawal as well as idealization/devaluation of relationships (8). In more general terms, *impairment in interpersonal functioning* has been identified as one of the core features of psychopathology in BPD, alongside *affect dysregulation* and *behavioral dysregulation* (in particular impulsivity) (9). We reasoned that if we could illuminate how BPD traits give rise to specific pathological disturbances of the sense of commitment, this may also help us to understand the cognitive and motivational processes leading to impairments of interpersonal functioning in BPD.

Crucially, we opted not to investigate a clinical population for the current study. This was because we were primarily interested in the ways in which BPD traits may influence the sense of commitment generally—rather than the experiences of the clinical population specifically. Indeed, individuals with a BPD diagnosis often meet DSM criteria for other psychiatric conditions (e.g., major depression, anxiety disorders), as well as adverse childhood experiences (e.g., sexual abuse, neglect and abuse) (10), which would be difficult to disentangle from the effects of particular personality traits. Nevertheless, the non-clinical population with BPD traits has recently also generated research interest. It was shown that individuals with BPD traits show deficits in emotional understanding and management of both own and other's emotions (11, 12). Accordingly, we can expect to observe the difference in the emotional functioning of people with BPD traits even without the presence of diagnosis of BPD. We therefore recruited participants from the general population, and asked them (after the experiment) to complete the Five-Factor Borderline Inventory [FFBI-SF; (13)] to assess their BPD traits. Their responses to the FFBI-SF measure enabled us (as described below) to divide individuals into two groups according to their level of BPD traits (in accordance with the dimensional approach): High BPD and Low BPD.

By presenting our participants with vignettes describing situations in which every day implicit or explicit commitments were violated, and asking them to answer questions concerning their perceptions, interpretations, and affective and behavioral responses to those situations, we were able to probe several distinct hypotheses about how BPD traits may give rise to disturbances of the sense of commitment, which we will now introduce in turn.

**Hypothesis 1 (H1):** *High BPD individuals expect others to be less committed to joint activities*. H1 is motivated by the finding that individuals with BPD exhibit biases in attributional style [see (14, 15); for a review]. For instance, individuals with BPD have been found to exhibit biases in interpretation, evaluating others' intentions and actions as negative and malevolent (16, 17). Moreover, they also report being less trustful of others; besides investing less when playing the role of the investor in trust games (18), BPD individuals also expected the trustees to return a smaller portion of the money (19).
**Hypothesis 2 (H2):** *High BPD individuals calibrate their social expectations and interpretations less as a function of subtle situational factors, such as the investment of effort in a joint activity and the degree of coordination*. H2 is motivated by the finding that individuals with BPD tend to exhibit dichotomous thinking–i.e., shifting between extreme positive or negative evaluations of others, which may preclude subtle adjustments of expectations in light of a changing evidence base (20, 21).
**Hypothesis 3 (H3):** *High BPD individuals exhibit heightened emotional responses to perceived or anticipated violations of commitment*. H3 is motivated by the strong association of BPD with negative affect, both in terms of intensity and reactivity, as assessed by self-report and psychophysiological measures [see (22) for a review]. For instance, when playing the Cyberball game, individuals with BPD reported more intense rejection-related negative emotions than healthy controls not only in the exclusion condition when they rarely received the ball, but also in the inclusion condition (23, 24). Only in the over-inclusion condition where they received more ball passes than the other players did BPD individuals report comparable levels of negative emotions as healthy controls in the inclusion condition, suggesting that even socially inclusive contexts elicit rejection-related feelings in BPD (23).
**Hypothesis 4 (H4):** *High BPD individuals exhibit non-adaptive behavioral responses to perceived or anticipated violations of implicit commitment*. H4 is motivated by the fact that impairment in interpersonal functioning, which has been identified as one of the core features of BPD psychopathology (9), has been related to a lack of coping strategies targeting the regaining and maintenance of social relationships (25). For a striking illustration of this, consider a recent study by King-Casas et al. (26) using a trust game. King-Casas and colleagues observed that when an investor initiated a rupture in cooperation by sending a small investment, healthy control trustees were able to repair this rupture by returning a large sum of money to signal their trustworthiness to the investor. This so-called coaxing behavior served to restore trust, resulting in higher investments in four subsequent rounds. BPD trustees, in contrast, did not employ this coaxing strategy, and responded to low investments with equally low repayments. Over the course of 10 rounds, this pattern resulted in decreasing investments in dyads involving BPD trustees, i.e., in a breakdown in cooperation.

## Methods

For all three experiments, we used *SurveyMonkey* to implement a web-based observational paradigm using a between-subjects design, and participant recruitment was conducted via Amazon Mechanical Turk (M-Turk). Since each participant gave only one judgment for each test question, and since online experiments produce greater variability than lab-based experiments, we expected a high variability in our dependent variables. We therefore opted for a large sample size: as in the original study on which experiment 1 was based (6), we aimed for 100 participants per group–i.e., in both Experiments 1, 2, we aimed for 400 participants (4 conditions, 100 per group); in Experiment 3 we aimed for 200 participants (2 conditions, 100 per group); Anticipating that roughly 10–20% would either fail to complete the questions or respond incorrectly to the control question, and that the randomization procedure would lead to unequal numbers of participants in each condition, we therefore requested 500 participants for Experiments 1, 2, and 220 participants for Experiment 3. We also included data from those participants who had already begun the experiment when M-Turk registered that this number had been reached. All three experiments were conducted in accordance with the Declaration of Helsinki and was approved by the Humanities & Social Sciences Research Ethics Sub-committee (HSSREC) at the University of Warwick. All participants received a monetary compensation of $0.50.

In all three experiments, participants' BPD traits were assessed using the short form of the Five-Factor Borderline Inventory [FFBI-SF; (13)]. The FFBI-SF is a 48-item self-report measure which assesses BPD traits based on the Five Factor Model [FFM; (27)] of general personality. The measure has a high degree of internal consistency, convergent, discriminant, and incremental validity. Internal consistency for the total score in this sample was Cronbach's α = 0.98. Since we had no a priori hypotheses as to whether the relationship between BPD traits and our dependent variables would be linear, or whether any differences might be driven just by individuals with particularly high levels of BPD traits, we opted for a variance-analytical approach comparing subjects high and low in BPD features. The BPD trait variable was therefore dichotomized using a median split within each experiment.

For all analyses, we set the significance level at *p* = 0.05. We report exact *p*-values except where *p* < 0.001, in which case we report *p* < 0.001. The *p*-values were not adjusted, as all analyses below were planned comparisons.

### Experiment 1

Experiment 1 builds upon an earlier study in which Michael et al. (6) presented participants with videos of joint actions with either a low degree of coordination or a high degree of coordination, and asked participants to judge whether and for how long the observed agents would resist a tempting outside option and remain engaged in the joint action. In addition to replicating the findings of this earlier study, Experiment 1 was designed to test H1, H2, H3, and H4. We predicted that participants with a high level of BPD traits, when imagining themselves in the scenario presented in the video, would expect less commitment from their partner (H1), that their expectations would vary less as a function of the degree of coordination (H2), that they would indicate more intense negative emotional reactions if their partner disengaged from the joint action and more intense positive emotions if their partner resisted the temptation to disengage (H3), and that they would be more likely to respond to a commitment violation by withdrawing from the relationship rather than smoothing things over.

#### Participants

After excluding 59 participants because they either did not complete all of the questions, or incorrectly answered the control question, the final dataset included 537 non-clinical adults (251 women) between the ages 18 and 77 years (*M* = 37.20 years, *SD* = 13.35 years) (see Data Sheet 2). The BPD trait variable was dichotomized using a median split (*Mdn* = 89.00), and participants were divided into two groups: Low BPDt (*n* = 272, *M* = 64.65, *SD* = 12.24) and High BPDt (*n* = 265, *M* = 134.87, *SD* = 33.48). Using *SurveyMonkey*'s random assignment feature, participants were randomly assigned either to a High Coordination condition or a Low Coordination condition, creating four groups in total. See Table 1 below for the descriptive statistics for each group. Note however, that there was a significant effect of group on age, *F*_(3, 533)_ = 17.18, *p* < 0.001, *partial* η^2^ = 0.09, as well as a significant association between group and gender, *X*_(3)_ = 15.96, *p* = 0.001. Therefore, age and gender were included as covariates in the analyses below.

**Table 1:** Descriptive statistics for comparison groups in experiment 1.

**Groups**	***n* per group (*n* women)**	***M*_age_ (*SD*_age_)**	***M_*BPDt*_ (SD_*BPDt*_)***
H_Coordination_ H_BPDt_	125 (69)	36.73 (11.09)	123.04 (25.41)
H_Coordination_ L_BPDt_	173 (87)	40.60 (13.04)	64.49 (12.22)
L_Coordination_ H_BPDt_	140 (46)	31.57 (9.58)	145.43 (36.26)
L_Coordination_ L_BPDt_	99 (49)	39.99 (13.36)	64.94 (12.34)

#### Material and procedure

After giving her or his informed written consent and providing basic demographic information (gender and age), each participant performed one trial. At the beginning of the trial, participants were asked to read the following brief text and to imagine the situation described therein:

*You had some renovation work done on your house recently. This morning, you decided to clean up the pile of sand left over by the renovation work as shown in the picture below. You expect it to take about an hour. As you will see in a very brief video, your neighbor Thomas needs to get home and finds his way blocked by the pile of sand, and decides to help for a bit*.

Next, they viewed one of two videos, depending on experimental condition (see Data Sheet 4)^1^. In the high coordination condition, the two agents form a chain, with one agent filling a bucket and passing it to the other agent in the chain. In the Low Coordination condition, the two agents work in parallel.

In each condition, the process is repeated twice once the helper begins–i.e., the agents either exchange the buckets twice or walk past each other twice. The videos were approximately 40 s in length. When, after 40 s, the video stopped, participants were presented with the following questions, always in this order:

- The “perceived commitment question”: How long would you expect Thomas to continue to help? (“Not at all,” “for a few minutes,” “until about half the sand is cleaned up,” “until most of the sand is cleaned up,” “until the job has been completed”).
- The “gratitude question”: How would you feel if Thomas continued helping until all the sand had been cleaned up? (5-point scale from not grateful at all–he is my neighbor so he should help to highly grateful and touched by his kindness; 1–5).
- The “annoyance question”: If Thomas' phone rang and he took the call, how would you feel? (6-point scale from not at all annoyed to highly annoyed; 1–5).
- The “withdrawal question”: If Thomas took the phone call, how likely would you be to help him in the future? (5-point scale from highly likely to highly unlikely; 1–5).
- The control question: What did you and Thomas use to remove the sand pile? (shovels, buckets, garden scoops, or spades).

For the perceived commitment question, we predicted that participants would expect the agent to continue helping for a longer period in the High Coordination condition than in the Low Coordination condition. We also predicted that participants in the High BPDt group would give lower estimates than participants in the Low BPDt group (H1), and that their responses would differ less between the High Coordination and the Low Coordination conditions (H2). For the gratitude question, we predicted that they would respond to the gratitude question by indicating less gratitude, because they would be less inclined to adjust their interpretation of the other agent's intentions and attitudes in light of a changing evidence base (H2). We also predicted that they would respond to the annoyance question by indicating more intense negative emotions if their partner disengaged from the joint action (H3). Finally, we predicted that their responses to the withdrawal question would reveal a greater tendency to respond to a commitment violation by withdrawing from the relationship rather than smoothing things over (H4).

#### Results and discussion

For the control question, five participants (2 men, 2 women, 1 prefer not to say) did not correctly indicate “*buckets*” as the equipment used to remove the sand pile. There was no significant group difference between those who answered correctly on the control question and those who did not on the following variables: age, BPDt, and the perceived commitment, gratitude, annoyance, and withdrawal questions (*ps* = 0.33 −0.83).

We ran ANCOVAs for the (1) perceived commitment, (2) gratitude, (3) annoyance, and (4) withdrawal questions, controlling for age and gender for each analysis. The results are presented in Table 2.

**Table 2:** Analyses for experiment 1.

**DV**	**Main/interaction effects**	***F***	***df***	***partial* η2**
Perceived commitment	Condition	23.47^***^	(1,531)	0.04
	BPDt groups	6.21^*^	(1,531)	0.01
	Condition × BPDt groups	0.01	(1,531)	<0.001
Gratitude	Condition	10.63^**^	(1,531)	0.02
	BPDt groups	15.13^***^	(1,531)	0.03
	Condition × BPDt groups	1.09	(1,531)	<0.01
Annoyance	Condition	8.85^**^	(1,531)	0.02
	BPDt groups	30.63^***^	(1,531)	0.06
	Condition × BPDt groups	17.52^***^	(1,531)	0.03
Withdrawal	Condition	0.06	(1,531)	<0.001
	BPDt groups	7.17^**^	(1,531)	0.01
	Condition × BPDt groups	2.78	(1,531)	<0.01

* Are significant at p < 0.05.

** Are significant at p < 0.01.

*** Are significant at p < 0.001.

*Age and gender were included as covariates in each analysis. Only the main and interaction effects of interest are presented in this table*.

**Figure 1:**
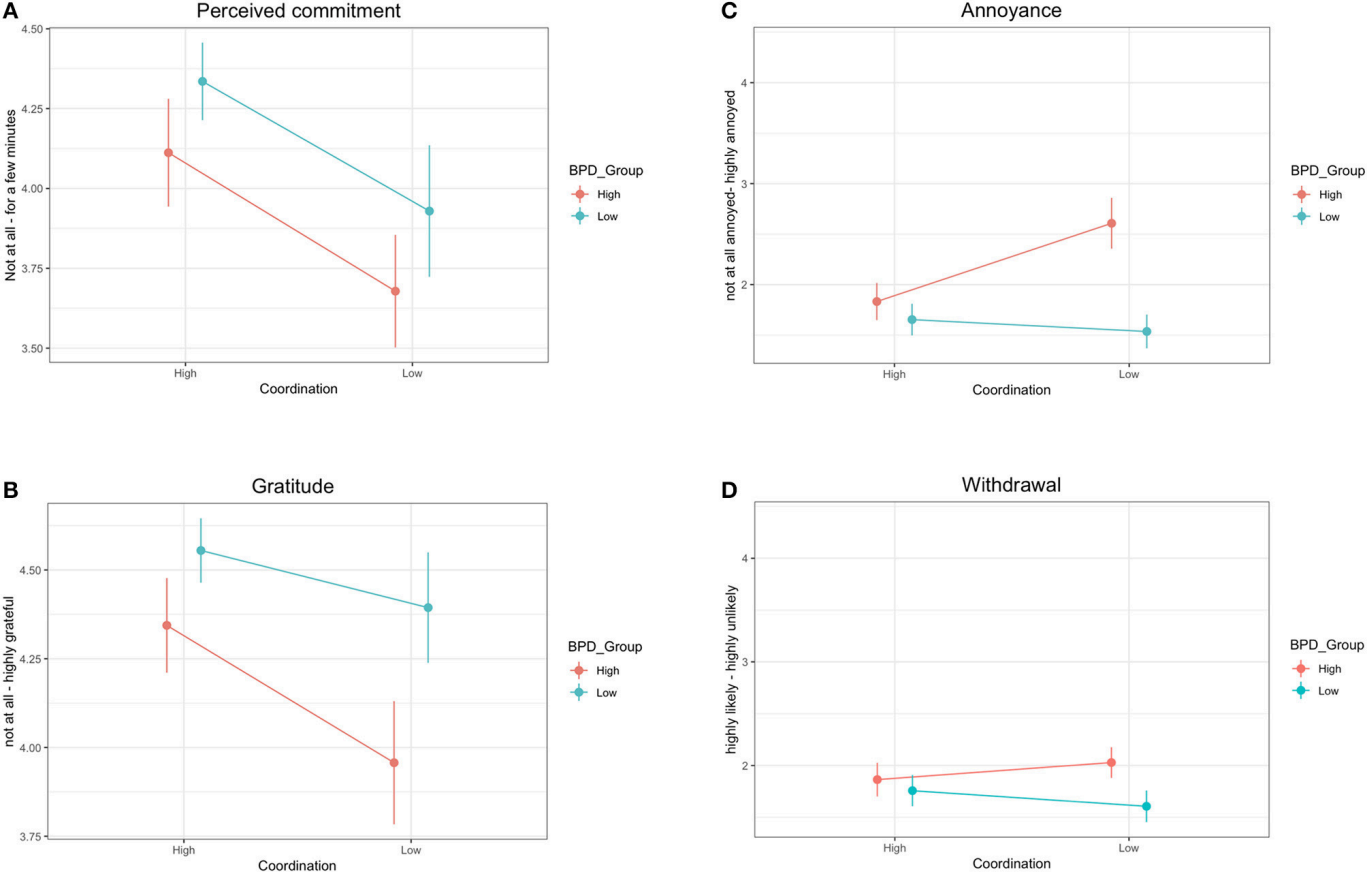
Results to the four test questions for the High BDP and Low BPD groups in the High. Coordination and Low Coordination conditions. Error bars represent the confidenceintervals. **(A)** shows participants' responses to the perceived commitment question (“How long would you expect Thomas to continue to help?”). **(B)** shows responses to the gratitude question (“How would you feel if Thomas continued helping until all the sand had been cleaned up?”). **(C)** shows responses to the annoyance question (“If Thomas' phone rang and he took the call, how would you feel?”). **(D)** shows responses to the withdrawal question (“If Thomas took the phone call, how likely would you be to help him in the future?”).

For the perceived commitment question (see Figure 1A), results showed a significant main effect of coordination, with participants giving higher estimates in the High Coordination Condition (*M* = 4.24, *SD* = 0.88) than the Low Coordination condition (*M* = 3.78, *SD* = 1.05). This replicates the finding reported in the original study (6). There was also a significant main effect of BPD group, with participants in the High BPDt group giving lower estimates (*M* = 3.88, *SD* = 1.03) than those in the Low BPDt group (*M* = 4.19, *SD* = 0.92). This corroborates our prediction, providing evidence for the hypothesis (H1) that individuals with high levels of BPD traits have low expectations about others' sense of commitment to joint activities. There was no significant interaction between coordination and BPD group. This was not consistent with our prediction that responses in the High BPDt group would differ less between the High Coordination and the Low Coordination conditions (H2).

Next, for the gratitude question (see Figure 1B), results revealed a significant main effect of coordination, with participants feeling more grateful in the high coordination condition (*M* = 4.47, *SD* = 0.68) than the low coordination condition (*M* = 4.14, *SD* = 0.96). There was also a significant main effect of BPD group, with participants in the High BPDt group feeling less grateful (*M* = 4.14, *SD* = 0.93) than those in the Low BPDt group (*M* = 4.50, *SD* = 0.68). This corroborates our prediction, providing support for the hypothesis (H2) that individuals with high levels of BPD traits are less inclined to adjust their interpretations of others' intentions and attitudes in light of a changing evidence base. There was no significant interaction between coordination and BPD group.

For the annoyance question (see Figure 1C), results revealed a significant main effect of coordination, with participants feeling more annoyed in the low coordination condition (*M* = 2.16, *SD* = 1.38) than in the high coordination condition (*M* = 1.73, *SD* = 1.04). This replicates the finding reported in the original study (6). There was also a significant main effect of BPD group, with participants in the High BPDt group feeling more annoyed (*M* = 2.24, *SD* = 1.36) than those in the Low BPDt group (*M* = 1.61, *SD* = 0.97). This corroborates our prediction, providing evidence in support of the hypothesis (H3) that High BPDt individuals exhibit heightened emotional responses to perceived or anticipated violations of commitment. There was a significant interaction between condition and BPD group. To explore this interaction, we ran two ANCOVAs for (1) high coordination and (2) low coordination, controlling for age and gender. For high coordination, participants in the high BPDt group (*M* = 1.83, *SD* = 1.04) were not significantly more annoyed than those in the low BPDt group (*M* = 1.65, *SD* = 1.04), *F*_(1, 294)_ = 1.53, *p* = 0.22, *partial* η^2^ = 0.01. For low coordination, however, participants in the high BPDt group (*M* = 2.61, *SD* = 1.51) reported being significantly more annoyed than those in the low BPDt group (*M* = 1.54, *SD* = 0.84), *F*_(1, 235)_ = 30.31, *p* < 0.001, *partial* η^2^ = 0.11. The plot (see Figure 1C) indicates that participants in the High BPDt group reported particularly high levels of annoyance in the low coordination condition (see section General Discussion).

Finally, for the withdrawal question (see Figure 1D), results did not reveal a significant difference between the low coordination condition (*M* = 1.85, *SD* = 0.86) and the high coordination condition (*M* = 1.80, *SD* = 0.97). There was a significant main effect of group, with participants in the High BPDt group reporting less willingness (*M* = 1.95, *SD* = 0.91) than those in the Low BPDt group (*M* = 1.70, *SD* = 0.93). This corroborates our prediction, supporting the hypothesis that individuals with high levels of BPD traits exhibit a greater tendency to respond to perceived commitment violations by withdrawing from relationships rather than smoothing things over (H4). There was no significant interaction between condition and BPD group, although we did observe a trend. As the plot in Figure 1D shows, High BPDt participants reported being especially inclined to withdraw in the low coordination condition, whereas this pattern was reversed for Low BPDt participants (see section General Discussion).

### Experiment 2

Experiment 2 extends the findings observed in Experiment 1 by manipulating a different situational factor, namely cost, instead of coordination. We hypothesized that the cost (in this case the effort cost) that one's partner has invested in a joint action modulates one's sense of being committed to remaining engaged and to resisting tempting alternative options. To test this, we presented participants with vignettes describing an everyday scenario in which one agent invests either a high degree of effort (High Cost condition) or a low degree of effort (Low Effort condition) to a joint action to which she and a second agent are implicitly committed, and the other agent then violates the implicit commitment by disengaging from the joint action. We predicted that participants would indicate a high degree of annoyance in the High Cost condition than in the Low Cost condition, and that they would be more inclined to judge that an apology would be appropriate. We also predicted that individuals with a high level of BPD traits would report higher levels of annoyance than participants with a low level of BPD traits, and that they would be more inclined to judge that an apology were in order (H3). We also predicted that the responses given to both questions by participants with high levels of BPD traits would differ less between the High Cost and the Low Cost conditions (H2).

#### Participants

After excluding 100 participants because they either did not complete all of the questions, or incorrectly answered the control question, the final dataset included 403 non-clinical adults (163 women) between the ages 21 and 68 (*M* = 34.88, *SD* = 10.91 years) (see Data Sheet 3). As in experiment 1, the BPD trait variable was dichotomized using a median split (*Mdn* = 93.00), and participants were divided into two groups: High BPDt (*n* = 200, *M* = 145.60, *SD* = 33.40) and Low BPDt (*n* = 203, *M* = 63.49, *SD* = 13.36). Using *SurveyMonkey*'s random assignment feature, participants were randomly assigned either to a High Cost condition or a Low Cost condition, creating four groups in total. See Table 3 below for the descriptive statistics for each group. There was no evidence for a significant association between group and gender, *X*_(3)_ = 6.80, *p* = 0.08. However, there was a significant effect of group on age, *F*_(3, 396)_ = 16.09, *p* < 0.001, *partial* η^2^ = 0.11. Therefore, age was included as a covariate in the analyses below.

**Table 3:** Descriptive statistics for comparison groups in experiment 2.

**Groups**	***n* per group (*n* women)**	***M*_age_ (*SD*_age_)**	***M_*BPDt*_ (SD_*BPDt*_)***	**BPDt range**
H_Cost_ H_BPDt_	107 (49)	30.58 (7.15)	144.21 (34.17)	137.00
H_Cost_ L_BPDt_	92 (36)	39.41 (12.89)	63.56 (12.72)	45.00
L_Cost_ H_BPDt_	90 (27)	32.29 (10.17)	147.29 (32.55)	117.00
L_Cost_ L_BPDt_	111 (51)	37.37 (10.71)	63.43 (13.93)	45.00

#### Materials and procedure

After giving her or his informed written consent and providing basic demographic information (gender and age), each participant performed one trial. At the beginning of the trial, participants were asked to read one of two versions of the following text (depending on the experimental condition) and to imagine the situation described therein:

##### High cost/low cost condition

You and Pam used to work in the same office on the 5th floor, until you were moved to (a 1st floor office / a different office down the hall on the same floor) 1 year ago. Every day for the past 3 years, you and Pam have spent your afternoon coffee break sitting out on the 5th floor balcony and chatting, though you never agreed to start doing this. After you moved to the new office (down on the 1st floor/down the hall), you nevertheless continued to (walk up to the same balcony on the 5th floor/walk down the hall to the balcony) every day to spend the coffee break with Pam, even though the balcony is (five flights of stairs up/down the hall) from your new office. The sequence is broken when 1 day you walk (all the way up the five flights of stairs / down the hall to the balcony) and wait for Pam during the coffee break, but she doesn't turn up.

Participants were then presented with the following two questions, each on a separate screen but always in this order:

- The “apology question”: To what extent would you agree Pam owes you an apology? (6 point scale ranging from disagree strongly to agree strongly; 0–5)
- The “annoyance question”: If Pam did not apologize or offer any explanation, how annoyed would you be? (6 point scale ranging from not at all annoyed to highly annoyed; 0–5)
- Control question: In the scenario described above, where is it that you and Pam spend the coffee break? (in the lounge, in the cafeteria, on the balcony).

We expanded the scale from 5 to 6 points in order to remove the middle option, thereby compelling participants to choose an option on either the upper or the lower half of the scale. We hypothesized that individuals with high levels of BPD traits are prone to heightened emotional responses to perceived violations of commitment (H3). We therefore predicted that participants in the High BPDt group would agree more strongly with the apology question, and that they would report higher degrees of annoyance in response to the annoyance question.

The cost manipulation (High Cost condition—Low Cost condition) was designed to probe the hypothesis that individuals with high levels of BPD traits are less sensitive to subtle situational factors which modulate the degree of commitment (H2). We predicted that participants in both the High BPDt and the Low BPDt groups would agree more strongly with the apology question in the High Cost condition than in the Low Cost condition, and that they would report higher degrees of annoyance in response to the annoyance question in the High Cost condition than in the Low Cost condition. We also predicted that an interaction for both test questions such that responses given by participants in the High BPDt group would differ less between the High Cost condition and the Low Cost condition than the responses of participants in the Low BPDt group.

#### Results and discussion

For the control question, 25 participants (16 men, 9 women) did not correctly indicate “*on the balcony*” as where the reader and Pam would spend the coffee break. There was no significant group difference between those who answered correctly on the control question and those who did not on age (*p* = 0.40). However, compared to those who answered correctly, participants who answered incorrectly on the control question reported significantly higher BPDt (*M*_balcony_ = 100.83, *SD*_balcony_ = 46.62 vs. *M*_other_ = 167.45, *SD*_other_ = 32.88), expected the apology more strongly (*M*_balcony_ = 2.92, *SD*_balcony_ = 1.48 vs. *M*_other_ = 4.12, *SD*_other_ = 1.33), and reported feeling more annoyed (*M*_balcony_ = 2.86, *SD*_balcony_ = 1.33 vs. *M*_other_ = 4.04, *SD*_other_ = 1.31) (*ps* ≤ 0.001).

We ran ANCOVAs for the (1) apology, and (2) annoyance questions, controlling for age and gender for each analysis. The results are presented in Table 4.

**Table 4:** Analyses for experiment 2.

**DV**	**Main/interaction effects**	***F***	***df***	***partial* η2**
Apology	Condition	9.42^**^	(1,394)	0.02
	BPDt groups	11.11^**^	(1,394)	0.03
	Condition × BPDt groups	3.27	(1,394)	<0.01
Annoyance	Condition	3.90^*^	(1,394)	0.01
	BPDt groups	14.85^***^	(1,394)	0.04
	Condition × BPDt groups	2.48	(1,394)	<0.01

* Are significant at p < 0.05.

** Are significant at p < 0.01.

*** Are significant at p < 0.001.

*Age and gender were included as covariates in each analysis. Only the main and interaction effects of interest are presented in this table*.

**Figure 2:**
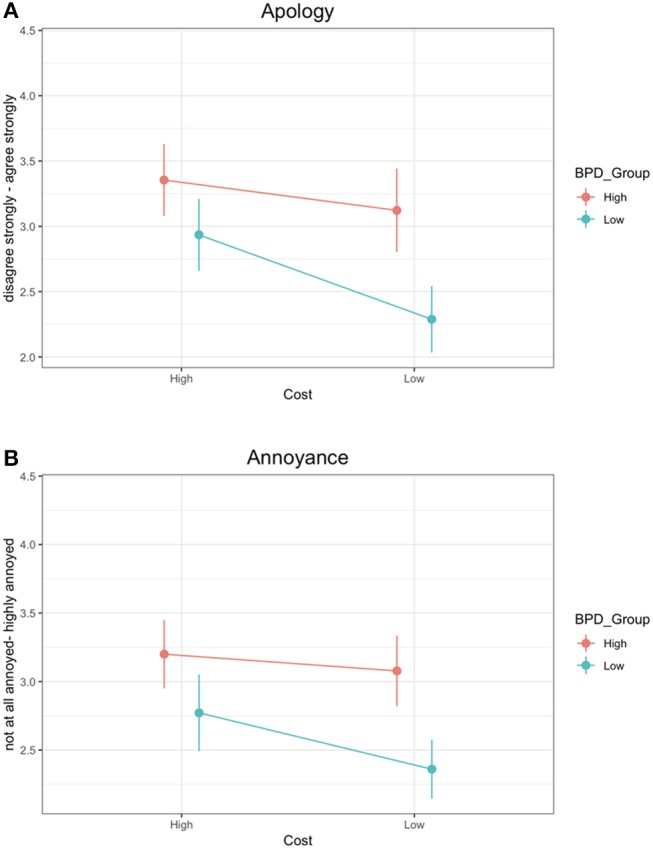
Results from the two test questions for the High BDP and Low BPD groups in the High Cost and Low Cost conditions. Error bars represent the confidence intervals. **(A)** shows responses to the apology question (“To what extent would you agree Pam owes you an apology?”). **(B)** shows responses to the annoyance question (“If Pam did not apologize or offer any explanation, how annoyed would you be?”).

For the apology question (see Figure 2A), results revealed a significant main effect of condition, with participants expecting the apology more strongly in the high cost condition (*M* = 3.16, *SD* = 1.41) than in the low cost condition (*M* = 2.66, *SD* = 1.49). This is consistent with our prediction, and confirms that participants tend to interpret the scenario in the High Cost condition as involving a higher level of commitment than the scenario in the Low Cost condition. There was also a significant main effect of BPD group, with participants in the High BPDt group more confidently asserting that an apology would be appropriate (*M* = 3.25, *SD* = 1.49) than those in the Low BPDt group (*M* = 2.58, *SD* = 1.38). This corroborates our prediction, providing evidence in support of the hypothesis (H3) that High BPDt individuals exhibit heightened emotional responses to perceived violations of commitment. There was no significant interaction between condition and BPD group. This is not consistent with the prediction we made based on the hypothesis that individuals with high levels of BPD traits are less sensitive to subtle situational factors which modulate the degree of commitment (H2).

Then, for the annoyance question (see Figure 2B), the analysis revealed a significant main effect of condition, with participants reported feeling more annoyed in the high cost condition (*M* = 3.00, *SD* = 1.35) than in the low cost condition (*M* = 2.68, *SD* = 1.23). There was also a significant main effect of BPD group, with participants in the High BPDt group feeling more annoyed (*M* = 3.15, *SD* = 1.28) than those in the Low BPDt group (*M* = 2.55, *SD* = 1.26). This corroborates our prediction, providing evidence in support of the hypothesis (H3) that High BPDt individuals exhibit heightened emotional responses to perceived violations of commitment. There was no significant interaction between condition and BPD group. This is not consistent with the prediction we made based on the hypothesis that individuals with high levels of BPD traits are less sensitive to subtle situational factors which modulate the degree of commitment (H2).

### Experiment 3

In Experiment 3, we aimed to extend the findings from Experiments 1 and 2 by investigating how individuals with high levels of BPD traits evaluate and respond to scenarios in which *explicit* commitments are violated. On the basis of our hypothesis that individuals with high levels of BPD traits have more negative expectations regarding others' sense of commitment to joint activities (H1), we reasoned that they may be less inclined than individuals with low levels of BPD to assume that there was a good reason for the person's failure to honor the commitment. We also reasoned that they may be less willing or able to adapt their expectations and evaluations in light of circumstances (H2), they may apply explicit norms more rigidly, and therefore be less forgiving over minor violations of explicit commitments (H3, H4). To test this, we presented participants with a vignettes describing an everyday scenario in which two neighbors make an agreement to meet for coffee, but one of them does not show up.

#### Participants

After excluding 26 participants because they did not complete all of the questions, the final dataset included 190 non-clinical adults (74 women) between the ages 21 and 71 (*M* = 34.52, *SD* = 11.07 years), all of whom were fluent English speakers (see Data Sheet 4). As in experiments 1 and 2, the BPD trait variable was dichotomized using a median split (*Mdn* = 86.91), and participants were divided into two groups: High BPDt and Low BPDt. See Table 5 below for the descriptive statistics for each group. There was no evidence for a significant association between group and gender, *X*_(1)_ = 0.09, *p* = 0.77. However, there was a significant effect of group on age, *t*_(163.53)_ = 4.58, *p* < 0.001, *d* = 0.72. Therefore, age was included as a covariate in the analyses below.

**Table 5:** Descriptive statistics for comparison groups in experiment 3.

**Groups**	***n* per group (*n* women)**	***M*_age_ (*SD*_age_)**	***M_*BPDt*_ (SD_*BPDt*_)***	**BPDt range**
H_BPDt_	95 (38)	31.45 (8.42)	127.64 (26.57)	104.00
L_BPDt_	95 (36)	38.60 (12.66)	60.84 (10.55)	38.00

#### Materials and procedure

BPD traits were again assessed via the short form of the Five-Factor Borderline Inventory [FFBI-SF; (13)]. The total score was calculated by summing the responses to all 48 items.

After giving her or his informed written consent and providing basic demographic information (gender and age), each participant performed one trial. At the beginning of the trial, participants were asked to read the following text and to imagine the situation described therein:

*You have just moved to a new neighborhood and meet your neighbor Kathrin on the street. She suggests that the two of you meet for coffee. You invite her over to your place the following morning. She agrees to come at 11 a.m. The next day, you are waiting for her and notice that it is already 11:15 a.m*.

Participants were then presented with the following questions, each on a separate screen but always in this order:

- The “likelihood question”: Based on your previous experiences with other people, how likely is it that she will come? (6-point scale ranging from extremely unlikely to extremely likely; 0–5).
- The “reasoning open question”: Based on your experiences with other people, list up to 3 reasons why you think she hasn't shown up.
- The “annoyance question”: Rate how you would feel for each of the reasons you listed (6-point scale ranging from not at all annoyed to highly annoyed; 0–5).
- The “rescheduling question”: For each of the reasons you listed, rate how interested you would be in rescheduling the coffee meet-up. (6-point scale ranging from not at all interested to highly interested; 0–5).
- The “feeling question”: How would you feel if Kathrin doesn't show up at all? (6-point scale ranging from not at all annoyed to highly annoyed; 0–5).

As in Experiment 2, we aimed to test the hypothesis that individuals with high levels of BPD traits are prone to heightened emotional responses to perceived violations of commitment (H3)–this time explicit commitment. We predicted that participants in the High BPDt group would give lower estimates in response to the likelihood question than participants in the Low BPDt group (H1). If participants in the High BPDt group have more negative expectations with regard to others fulfilling commitments (H1), then we should also expect them to give more negative responses to the reasoning open question.

The reasoning open question also served to control for interpretive biases in analyzing the responses to the annoyance question and the feeling question. If the inferences that participants in the High BPDt group drew about the reasons why Kathrin has not shown up were more negative than the reasons given by participants in the Low BPDt group, this difference may plausibly influence their responses to the annoyance question and the feeling question.

For the annoyance question, we predicted that participants in the High BPDt group would report higher degrees of annoyance (H3). Similarly, for the rescheduling question, we predicted that participants in the High BPDt would be less interested in rescheduling the meeting (H3, H4).

Similarly, for the feeling question, we predicted that participants in the High BPDt would report more negative feelings (H3).

#### Results and discussion

We ran ANCOVAs for the (1) likelihood, (2) reasoning, (3) annoyance, (4) rescheduling, and (5) feeling questions, controlling for age and gender for each analysis. The results are presented in Table 6.

**Table 6:** Analyses for experiment 3.

**DV**	**Main effects**	***F***	***df***	***partial η*2**
Likelihood	BPDt groups	2.97	(1,186)	0.02
Reasoning	BPDt groups	2.48	(1,186)	0.01
Annoyance	BPDt groups	10.75^**^	(1,186)	0.06
Rescheduling	BPDt groups	6.26^*^	(1,186)	0.03
Feelling	BPDt groups	5.07^*^	(1,186)	0.03

* Are significant at p < 0.05.

** Are significant at p < 0.01.

*Age and gender were included as covariates in each analysis. Only the main and interaction effects of interest are presented in this table*.

For the likelihood question, results revealed no significant differences in the reported likelihood that Kathrin will eventually show up between High BPDt (*M* = 3.73, *SD* = 1.05) and Low BPDt group (*M* = 3.94, *SD* = 0.97). This was not consistent with our prediction that the estimates of participants in the High BPDt group would be lower than those in the Low BPDt group (H1).

We coded the reasoning open question for the presence of negative responses. A score of 1 was assigned when an interpretation was negative (e.g., she doesn't want to come; she's not interested in me) and a score of 0 was assigned when no negativity was present in the interpretation (e.g., she forgot, she's running late). Responses were coded as “missing” if they were ambiguous/unclear, or irrelevant to the question (e.g., lazy, priority). Three coders (first author (JO), a co-author (AF), and a research assistant) each coded all the responses. All coders were blind to participants' BPD scores/group. A score (ratio between negative and neutral responses) was then calculated for each participant. To check for reliability, a two-way random effects ANOVA model was adopted, given that a random sample of 3 coders were selected and each of the 3 coders coded all the responses. Moreover, for greater reliability, mean ratings were used as the unit of reliability. Inter-rater reliability for the reasoning open question was ICC (2,3) = 86.

For the reasoning question, results revealed no significant differences in the emotional character of generated reasons between High BPDt (*M* = 0.32, *SD* = 0.46) and Low BPDt group (*M* = 0.23, *SD* = 0.41). This was again not consistent with our prediction that participants in the High BPDt group would give more negative responses regarding their expectations about others fulfilling commitments (H1).

**Figure 3:**
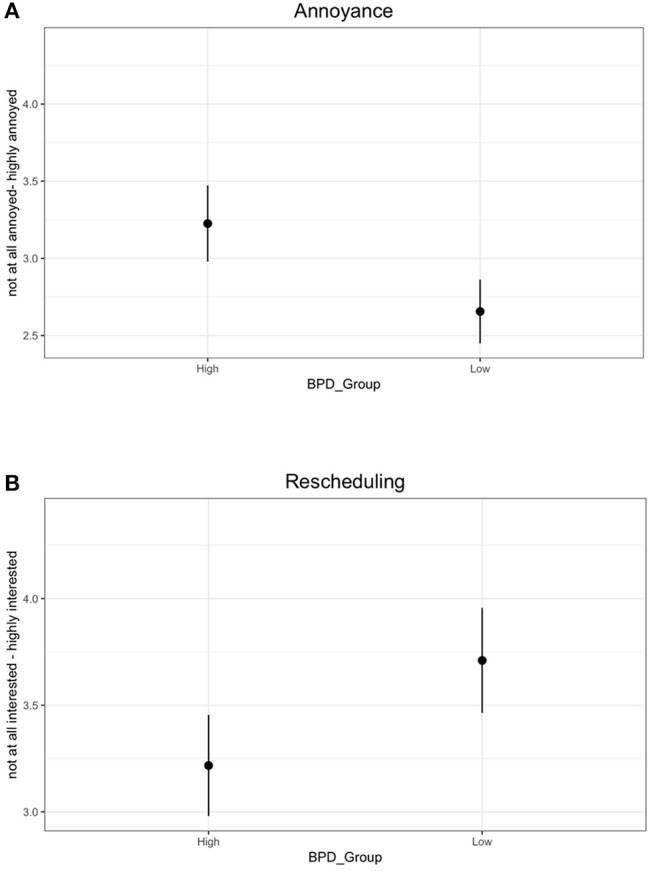
Results from the two test questions for the High BDP and Low BPD groups. Error bars represent the confidence intervals. **(A)** shows responses to the annoyance question (“Rate how you would feel for each of the reasons you listed”). **(B)** shows responses to the rescheduling question (“Rate how interested you would be in rescheduling the coffee meet-up”).

For the annoyance question, since participants were asked to rate how annoyed they would be for all 3 reasons they generated, the average score was calculated for each participant. There was a significant effect of BPD group on responses to the annoyance question, with participants in the High BPDt group feeling more annoyed (*M* = 3.19, *SD* = 1.12) than the participants from Low BPDt group (*M* = 2.61, *SD* = 1.08). This corroborates our prediction, providing support for the hypothesis (H3) that individuals with high levels of BPD traits exhibit heightened emotional responses to perceived or anticipated violations of commitment (see Figure 3A).

Next, for the interest in rescheduling question, since participants were asked to rate their interest in rescheduling the meeting separately for all 3 reasons they generated, again the average score was calculated. There was a significant effect of BPD group on participants' interest in rescheduling, with participants in the High BPDt group reporting less interest in rescheduling the meeting (*M* = 3.25, *SD* = 1.10) than participants in Low BPDt group (*M* = 3.74, *SD* = 1.30). This supports the hypothesis (H3) that individuals with high levels of BPD traits exhibit heightened emotional responses to perceived or anticipated violations of commitment (see Figure 3B). In addition, it also provides support for the hypothesis (H4) that High BPDt individuals exhibit non-adaptive behavioral responses to perceived or anticipated violations of commitment.

For the feeling question, results revealed a significant difference in the level of annoyance, with participants in the High BPDt feeling more annoyed if Kathrin did not show up at all (*M* = 4.51, *SD* = 1.18) than participants in the Low BPDt group (*M* = 4.00, *SD* = 1.38). Similarly to the annoyance question, this result supports the hypothesis (H3) that individuals with high levels of BPD traits exhibit heightened emotional responses to perceived or anticipated violations of commitment.

## General discussion

Our first hypothesis (H1) was that individuals with high levels of BPD traits are more pessimistic about others fulfilling their commitments than individuals with low levels of BPD traits. This is supported by Experiment 1: participants in the High BPDt group reported lower estimates of how long they would expect their neighbor to continue helping. In contrast to this, the findings from Experiment 3 revealed no significant differences between participants in the High BPDt group and participants in the Low BPDt group with respect to their expectations about a neighbor honoring an explicit commitment to show up to a casual social engagement. This pattern of findings suggests a qualification of H1: participants with high levels of BPD may be less inclined to expect others to act in accordance with an implicit sense of commitment, but may not be less inclined to expect others to honor explicit commitments.

We also aimed to test the hypothesis (H2) that individuals with high levels of BPD traits calibrate their social expectations and interpretations less as a function of subtle situational factors, such as the degree of coordination and the investment of effort in a joint activity. The findings from Experiments 1, 2 did not provide evidence in support of this hypothesis. In Experiment 1, participants in the High BPDt group, like participants in the Low BPDt group, had higher expectations regarding the neighbor's willingness to help in the High Coordination condition than in the Low Coordination condition. And indeed, the interaction that we observed for the annoyance question indicates that the difference between High and Low coordination made *more* of a difference for participants in the High BPDt group than for participants in the Low BPDt group. We may speculate (in line with H1) that this was because their default expectations were lower, so that in the absence of the additional cue to cooperation provided in the High Coordination condition, they were more likely to infer that the neighbor would entirely disengage from the joint activity and not resume helping after the phone call. Similarly, in Experiment 2, participants in the High BPDt group, like participants in the Low BPDt group, were more inclined to judge that an apology would be appropriate when an implicit commitment had been violated in the High Cost condition than in the Low Cost condition, and indicated a higher level of annoyance if no apology or explanation were offered.

We found clear support for the hypothesis (H3) that individuals with high levels of BPD traits exhibit heightened emotional responses to perceived or anticipated violations of commitment. In all three experiments, participants in the High BPDt group indicated a higher level of annoyance than participants in the Low BPDt group over the violation of an implicit (Experiments 1, 2) or an explicit (Experiment 3) commitment.

Similarly, our results provide unequivocal support for the hypothesis (H4) that individuals with high levels of BPD exhibit non-adaptive behavioral responses to perceived or anticipated violations of implicit commitment. Specifically, in Experiment 1, participants in the High BPDt group gave lower estimates than participants in the Low BPDt group of the likelihood of their helping the neighbor in the future, and participants in the High BPDt group in Experiment 3 reported less willingness to reschedule the casual meeting with their neighbor who failed to show up to the meeting.

Taken together, these findings suggest that high levels of BPD traits may give rise to a difficulty in adapting one's social expectations and behavior in light of interpersonal commitments and in a manner that is calibrated to the social norms in the community. In view of the importance of interpersonal commitment for healthy social relationships, this could be an important factor contributing to some of the interpersonal difficulties experienced by many individuals with high levels of BPD traits–e.g., conflicted relationships, difficulty trusting others, fear of abandonment, and patterns of overinvolvement/withdrawal as well as idealization/devaluation of relationships.

It is also important to acknowledge a number of limitations of the present research, which give us reason to be cautious in interpreting the results. Most importantly, we did not enquire whether participants had ever received a BPD diagnosis or were exposed to adverse childhoods experiences. Given that some of our participants may well have had BPD diagnoses, we cannot exclude the possibility that our findings were driven by these participants. It would be beneficial for further research investigating BPD traits to determine which (if any) participants have had clinical diagnoses and to include this factor in analyses. Accordingly, it should be highlighted that implications from the present research may not extend to the clinical population, and replication of the studies using clinical samples would be crucial for informing prevention and/or treatment efforts. Moreover, there are some methodological limitations relevant to crowdsourcing tools, such as Amazon's MTurk. For instance, although MTurk participants tend to produce reliable data when self-reporting on clinically relevant symptoms (e.g., depression, social anxiety), Shapiro et al. (28) raised concerns about symptom malingering, which is the tendency to fabricate psychiatric symptoms. Specifically, the authors found that three percent of their sample scored above the recommended cut-off (29) on the Infrequency-Psychopathology Scale, self-reporting a high frequency of symptoms that tend to be extremely rare. Nevertheless, such sites suggest that such sites are a useful resource for accessing subclinical (traits) and clinical populations. The prevalence rate of clinically relevant symptoms such as depression, general anxiety and trauma exposure were comparable to representative samples, while the prevalence of social anxiety symptoms, unemployment and substance abuse problems were inflated compared to the general population; this allows for access to a participant pool exhibiting the full range of symptoms that may be difficult to access with more conventional methods (e.g., undergraduate samples).

## Author contributions

All authors listed have made a substantial, direct and intellectual contribution to the work, and approved it for publication.

### Conflict of interest statement

The authors declare that the research was conducted in the absence of any commercial or financial relationships that could be construed as a potential conflict of interest.

## Footnotes

^1^We used the same videos as were used in the study on which this experiment was based (6). The videos are included in the Supplementary Material of that original publication.

## Supplementary material

Data Sheet 1 Supplementary procedure for experiment 1.

Data Sheet 2 Data from experiment 1.

Data Sheet 3 Data from experiment 2.

Data Sheet 4 Data from experiment 3.
