# Supplementary material for: The Sense of Commitment in Individuals With Borderline Personality Traits in a Non-clinical Population
Source: Front Psychiatry. 2018 Nov 6;9:519. doi: 10.3389/fpsyt.2018.00519 (PMC6232377; doi:10.3389/fpsyt.2018.00519)
Supplement: Data Sheet 1 — Supplementary procedure for experiment 1. [file Data_Sheet_1.docx]

**Supplementary** **Procedure**

**Experiment 1**

**Instructions**

Please imagine the following situation, and keep it in mind as you watch the brief video:

You had some renovation work done on your house recently. This morning, you decided to clean up the pile of sand left over by the renovation work as shown in the picture below. You expect it to take about an hour. As you will see in a very brief video, your neighbour Thomas needs to get home and finds his way blocked by the pile of sand, and decides to help for a bit.


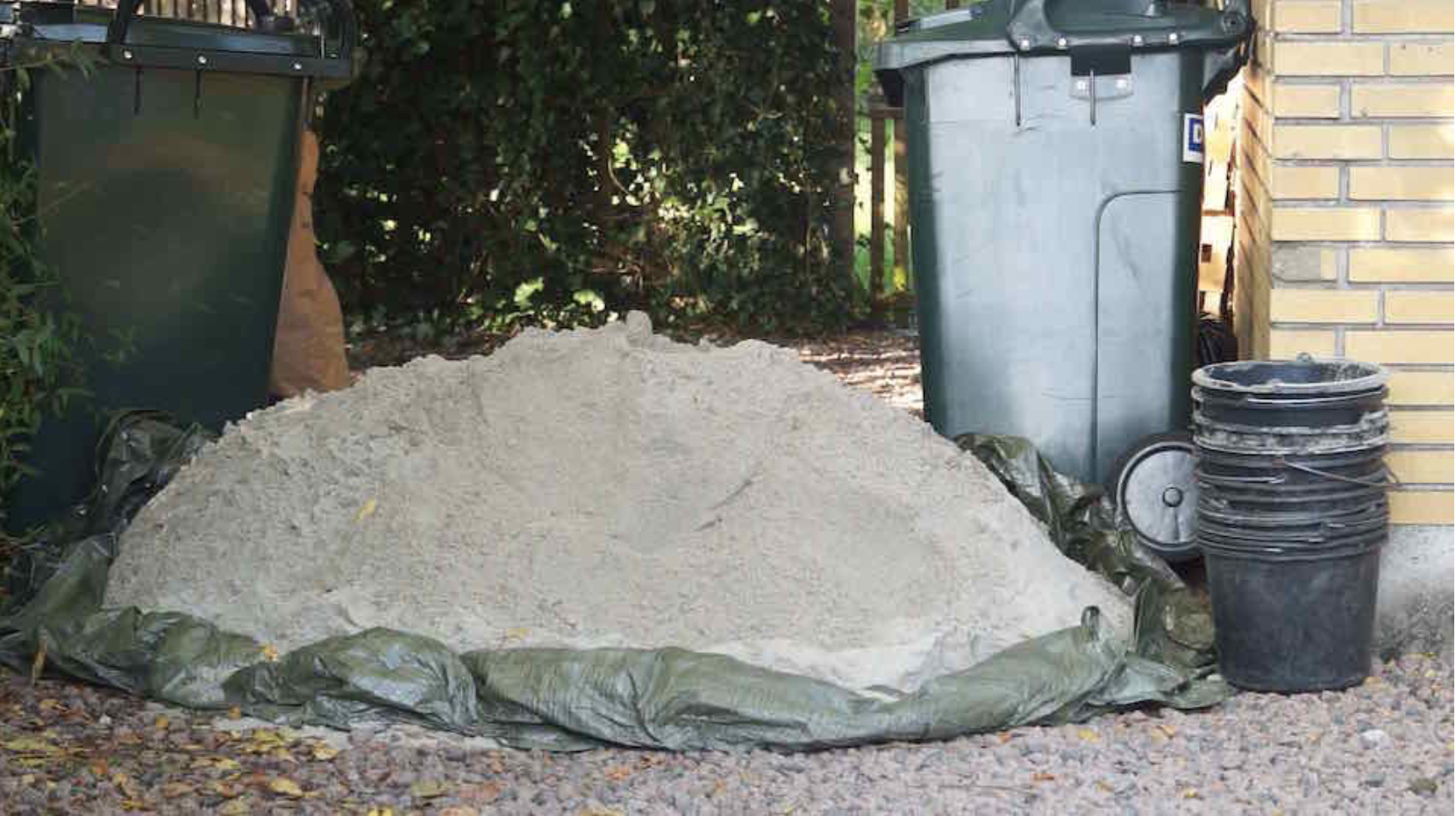


The video will take less than one minute, and then you will be asked a few questions about what you have seen. 
Please press ‘Next’ when you are ready to watch the brief video.

**Videos of the High Coordination (A1-A4) and the Low Coordination (B1-B4) conditions**


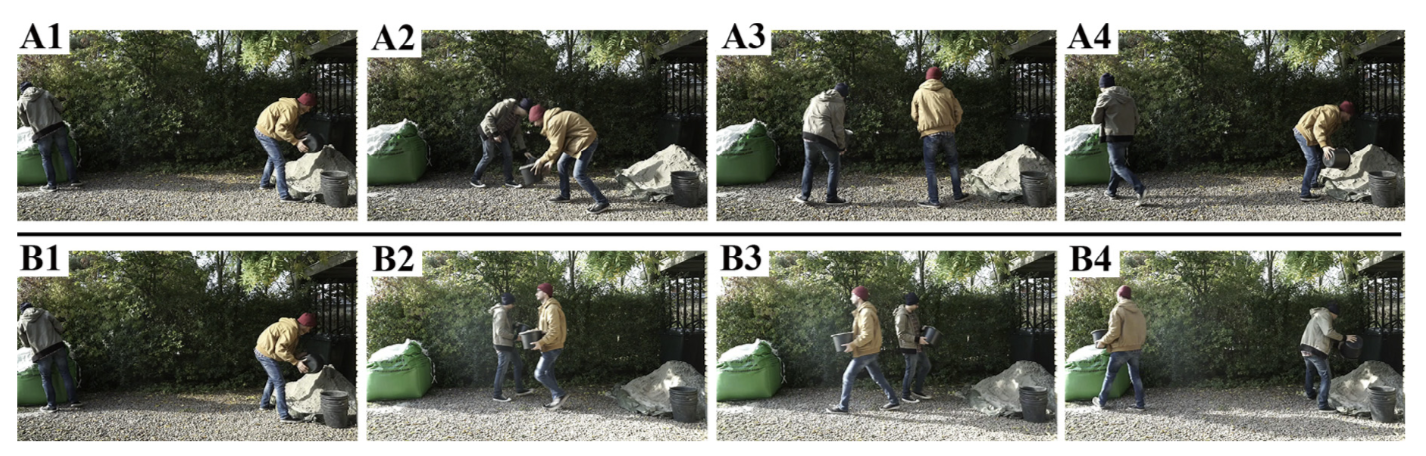


Depiction of the events presented in the videos viewed by participants in the high and low coordination conditions. A1-A4 are still frames from the video in the High Coordination condition, in which the two agents form a chain. B1-B4 are still frames from the video in the Low Coordination condition, in which the agents work in parallel.

**Questions**

1. How long would you expect Thomas to continue to help? (‘Not at all’, ‘for a few minutes’, ‘until about half the sand is cleaned up’, ‘until most of the sand is cleaned up’, ‘until the job has been completed’)
2. How would you feel if Thomas continued helping until all the sand had been cleaned up? (5-point scale from not grateful at all - he is my neighbour so he should help to highly grateful and touched by his kindness)
3. If Thomas’ phone rang and he took the call, how would you feel? (6-point scale from not at all annoyed to highly annoyed)
4. If Thomas took the phone call, how likely would you be to help him in the future? (5-point scale from highly likely to highly unlikely)
5. What did you and Thomas use to remove the sand pile? (shovels, buckets, garden scoops, or spades)

***Five-Factor Borderline Inventory (FFBI-SF, Deshong, Mullins-Sweatt, Miller, Widiger, & Lynam, 2015).***

Sample items:

I tend to act quickly without thinking things through.

I worry a lot about things that are out of my control.

At times, you have to be dishonest and manipulative to get what you need.

(Disagree strongly, Disagree a little, Neither agree nor disagree, Agree a little, Agree strongly)

**Experiment 2**

**Instructions**

There will be a short text describing everyday situations, which will be followed by some questions about the situation. Please press 'Next' when you are ready to go on to the text.

***High Cost Condition****:* You and Pam used to work in the same office on the 5th floor, until you were moved to a 1st floor office one year ago. Every day for the past three years, you and Pam have spent your afternoon coffee break sitting out on the 5th floor balcony and chatting, though you never agreed to start doing this. After you moved to the new office down on the 1st floor, you nevertheless continued to walk up to the same balcony on the 5th floor every day to spend the coffee break with Pam, even though the balcony is five flights of stairs up from your new office. The sequence is broken when one day you walk all the way up the five flights of stairs and wait for Pam during the coffee break, but she doesn’t turn up.

***Low Cost Condition****:* You and Pam used to work in the same office on the 5th floor, until you were moved to a different office on the same floor one year ago. Every day for the past three years, you and Pam have spent your afternoon coffee break sitting out on the balcony and chatting, though you never agreed to start doing this. After you moved to the new office, you nevertheless continued to walk over to the same balcony every day to spend the coffee break with Pam, as the balcony is just across the hall from your new office anyway. The sequence is broken when one day you walk over to the balcony and wait for Pam during the coffee break, but she doesn’t turn up.

**Questions**

1. To what extent would you agree Pam owes you an apology? (6-point scale ranging from disagree strongly to agree strongly)
2. If Pam did not apologize or offer any explanation, how annoyed would you be? (6-point scale ranging from not at all annoyed to highly annoyed)
3. In the scenario described above, where is it that you and Pam spend the coffee break? (in the lounge, in the cafeteria, on the balcony)

**Experiment 3**

**Instructions**

There will be a short text describing an everyday situation, which will be followed by some questions. Please press 'Next' when you are ready to go on to the text.

You have just moved to a new neighbourhood and meet your neighbour Kathrin on the street. She suggests that the two of you meet for coffee. You invite her over to your place the following morning. She agrees to come at 11 am.

The next day, you are waiting for her and notice that it is already 11:15 am.

**Questions**

1. Based on your previous experiences with other people, how likely is it that she will come? (6-point scale ranging from extremely unlikely to extremely likely)
2. Based on your experiences with other people, list up to 3 reasons why you think she hasn’t shown up.
3. Rate how you would feel for each of the reasons you listed (6-point scale ranging from not at all annoyed to highly annoyed)
4. For each of the reasons you listed, rate how interested you would be in rescheduling the coffee meet-up. (6-point scale ranging from not at all interested to highly interested)
5. How would you feel if Kathrin doesn’t show up at all? (6-point scale ranging from not at all annoyed to highly annoyed)
